# Quantification of endogenous and therapeutic IgG crossing the kidney barrier from bloodstream to urine

**DOI:** 10.3389/fphar.2025.1572739

**Published:** 2025-04-25

**Authors:** Barbara Eicher, Christoph Esslinger, Matthias Hillenbrand

**Affiliations:** Memo Therapeutics AG, Schlieren, Switzerland

**Keywords:** monoclonal antibody, pharmacokinetic, kidney epithelial barrier, kidney tubular lumen, urine, urothelia, therapeutic antibody, serum

## Abstract

This study investigates the biodistribution of a therapeutic antibody from serum to urine across the kidney endothelial barrier, contextualizing the findings with existing literature. Our analysis shows the quantitative correlation between serum levels of the intravenously administered antibody rituximab and its urinary concentration, indicating a predictable pharmacokinetic profile. The results align with previous quantitative studies on the biodistribution of endogenous and vaccination induced IgG between serum and urine, confirming that recombinant therapeutic IgG1 passes the kidney endothelial barrier and exhibits similar biodistribution to endogenous IgG. These insights may inform the determination of optimal dosages for therapeutic antibodies targeting the urothelium or renal epithelium.

## Introduction

The kidneys play a crucial role in maintaining serum levels of soluble proteins while excreting toxins, metabolic waste products, and excess ions and water. It is widely accepted that the filtration barrier, consisting of (1) podocyte pedicles, (2) the glomerular basement membrane, and (3) the fenestrated endothelium of glomerular capillaries, prevents proteins larger than 70 kDa from being excreted in the urine (Breshears and Confer, 2017). Given that the kidneys filter approximately 180 L of blood and over 2 kg of IgGs daily (Sand et al., 2015), the retention of IgGs in the bloodstream is essential.

It is commonly accepted that IgG does not pass the kidney barrier due to its size (Ryman and Meibohm, 2017). However, recent evidence suggests complex transport processes involving transcytosis allowing some serum IgG to be transported into the tubular lumen and subsequently be reabsorbed to some degree. In this context, it has been demonstrated that podocytes and proximal tubular epithelial cells, which line the lumen of the tubule, express the neonatal Fc receptor (FcRn), which actively transports IgG, along with albumin, via transcytosis (Haymann et al., 2000; Akilesh et al., 2008; Sarav et al., 2009; Sand et al., 2015; Dylewski et al., 2019). Several studies have shown that proximal tubular epithelial cells use FcRn to reabsorb IgG (Kobayashi et al., 2002; Rojas and Apodaca, 2002; Claypool et al., 2004; Sand et al., 2015; Lorentzen et al., 2023) from the filtrate and recycle it back into the bloodstream. Consequently, a portion of IgG passes through the kidney filtration barrier and enters the primary filtrate before being recycled into the blood or being partly excreted via urine (Burdon, 1971; Trinick and Laker, 1984; Reichert et al., 1997; Derré et al., 2020).

This transient presence of IgG in the tubular filtrate may play a crucial role in maintaining immunosurveillance in and around kidney tubular epithelial cells, which also serve as host cells or reservoirs for JC and BK polyomaviruses and CMV (Nickeleit et al., 2000; Li et al., 2010; Kant, 2020). In healthy individuals, these viruses are typically controlled by the immune system but may become reactivated during episodes of immunosuppression, e.g., upon transplantation.

In such cases, the biodistribution of IgG to the kidney tubular epithelium, ureter, and urothelium may be of interest for therapeutic interventions with monoclonal antibodies that target these viral infections or for other conditions affecting the kidneys, ureter or urothelium.

In the present study, we review quantitative data on the biodistribution of endogenous IgG in the serum and urine of human subjects, comparing it to data obtained upon administration with rituximab. Rituximab was chosen as a model antibody for therapeutically administered IgG due to the availability of donor samples and the presence of a sensitive, commercial anti-idiotypic ELISA for its detection.

## Methods

### Donors

Donors were lymphoma patients receiving rituximab. Serum and urine samples from each donor were collected during the same follow-up visit, all within 1 month after administration of rituximab.

Samples were acquired through Discovery Life Sciences (DLS), with appropriate institutional informed consent and in compliance with local ethical requirements.

### Quantification of rituximab in serum and urine by ELISA

The ab237640 Rituximab ELISA kit (Abcam) featuring a sensitive anti-idiotypic antibody to Rituximab as detection reagent was used according to the manufacturer’s instructions. Briefly, serum samples were diluted in assay buffer 400x, 800x, 1,600x, 3,200x, 6,400x. Urine samples collected in boritex^®^ tubes (Andwin Scientific, Simi valley, CA) and frozen at −18°C were used undiluted and diluted in assay buffer 4x, 10x, 20x, 40x and 100x. A serial dilution of a Rituximab standard was done in assay buffer according to the manufacturer’s recommendation, spanning from 3 to 300 ng/mL. Triplicates of sample and standard (100 µL each) were applied to the assay plate and incubated at room temperature, shaking for 1 h at 510 rpm. Subsequently, the plate was washed 3x with 300 µL wash buffer and tap-dried on a paper towel. Next, 100 µL HRP-conjugate was added to each well and incubated at room temperature for 1 h. After washing 3x with 300 µL wash buffer and tap-dried on a paper towel, 100 µL of TMB substrate was added and the plate incubated in the dark at room temperature for 15 min. Reaction was stopped with 100 µL stop solution and absorbance read at 450 nm and 650 nm (reference), using an Tecan Infinite M Nano plate reader.

The background subtracted data (Abs 450 – Abs 650 nm) was analyzed, calculating the average absorbance value for the blank control (0 ng/mL), followed by subtraction of this value from all samples. Values outside of the standard curve range were excluded from the calculation. A standard curve was generated in Graph Pad Prism 10, plotting the protein concentration (x-axis) against the corrected absorbance value (y-axis) and fitting the data with a four-parameter logistic fit. Based on this equation, the Rituximab concentration in each sample was calculated, compensating for the sample dilution.

### Preparation of data from published studies as summarized

IgG concentrations indicated in the original articles were used to calculate the percentage of urine concentration. For Derré et al. these concentrations were communicated to us by the authors.

Reichert et al. provide quantitative data about IgG in serum as g/L and in urine as milligram per day. In order to be able compare this with the serum IgG concentration, we assumed a daily urine quantity of 2 L (Raman et al., 2004). Thus, concentration values of urine IgG were calculated using the daily amount divided by 2 L. As an example, the average daily urinary IgG secretion of 98 mg/day (stable renal function) resulted in 49 mg/L urine which when related to the serum concentration of 5.3 g/L in serum yielded the data point 0.92% in our graph.

## Results and discussion

### Concentration of rituximab in serum and urine

Serum and urine samples taken at the same follow up visit from 5 patients undergoing rituximab treatment (Supplementary Table S1) at the time of sampling were obtained and levels of rituximab in serum and urine were determined (Table 1) by ELISA.

**TABLE 1:** Comparison of serum and urine rituximab levels in 5 donors receiving rituximab treatment as measured by a commercially available rituximab- specific ELISA assay.

	Antibody concentration		
Donor	Serum ± SD (µg/mL)	Urine ± SD (ng/mL)	Urine (%) of serum
1	59.4 ( ± 2.6)	0.9 ( ± 0.1)	0.002
2	53.1 ( ± 2.3)	16.2 ( ± 0.2)	0.03
3	45.1 ( ± 2.4)	1.7 ( ± 0.6)	0.004
4	24.6 ( ± 1.0)	3.6 ( ± 0.2)	0.015
5	57.8 ( ± 1.7)	2,453 ( ± 249)	4.2
Median	**53.1**	**3.6**	**0.015**
Geometric mean	**45.8**	**11.7**	**0.027**

The rituximab levels in treated donors are relatively widespread with a median percentage of 0.015% of the serum rituximab concentration present in the urine. This spread may well be due to a different stage of morbidity in the donors.

To contextualize our findings, we compared them with data from prior studies that examined serum and urine IgG levels. Some of these studies also compared urine IgG levels in healthy donors with those in patients with compromised renal or urothelial function or integrity (Burdon, 1971; Trinick and Laker, 1984; Reichert et al., 1997).

**FIGURE 1:**
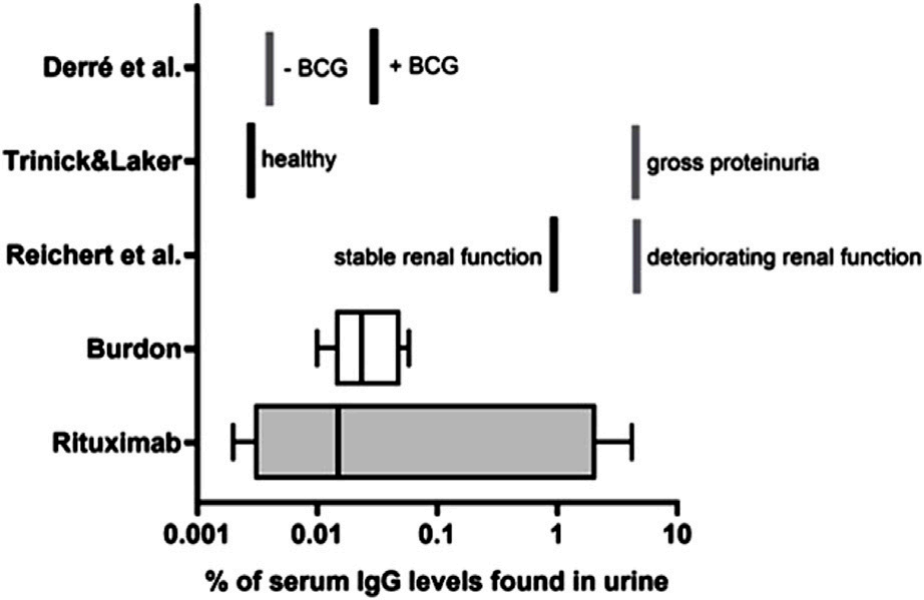
Urine IgG levels in relation to serum IgG *Rituximab:* i. v. Administered rituximab levels in serum and urine in 5 patients as measured by ELISA based on an anti-idiotypic antibody to rituximab (see also table 1 for individual levels). *Burdon* (Burdon, 1971): endogenous IgG levels in 4 healthy male and 4 healthy female subjects were quantified by radial diffusion in agar. *Reichert et al.* (Reichert et al., 1997) endogenous IgG levels in 22 patients with membranous nephropathy or nephrotic syndrome (deteriorating renal function) and normal renal function (stable renal function) were measured by immunonephelometry. Trinick & Laker (Trinick and Laker, 1984): endogenous IgG levels of 14 healthy adults (healthy) and six patients with severe proteinuria (gross proteinuria) were measured by ELISA. Derré et al. (Derré et al., 2020) IgG antibody titters in bladder cancer patients were monitored upon vaccination with a model antigen; 14 patients received *Bacillus* Calmette–Guérin (BCG), originally developed and used as a vaccine against tuberculosis and is now also used as a treatment for bladder cancer (+BCG) as treatment and 8 did not receive BCG (-BCG).

These data are summarized in Figure 1 below.

This comparison shows a good correlation between the different subject groups both across the previously published studies and with the rituximab treated subjects. The one outlier in the Rituximab group (right whisker of the graph), may be explained by an impaired kidney function of unknown etiology as it matches with the elevated levels found in patients with compromised kidney function (Reichert et al., 1997; Derré et al., 2020).

**FIGURE 2:**
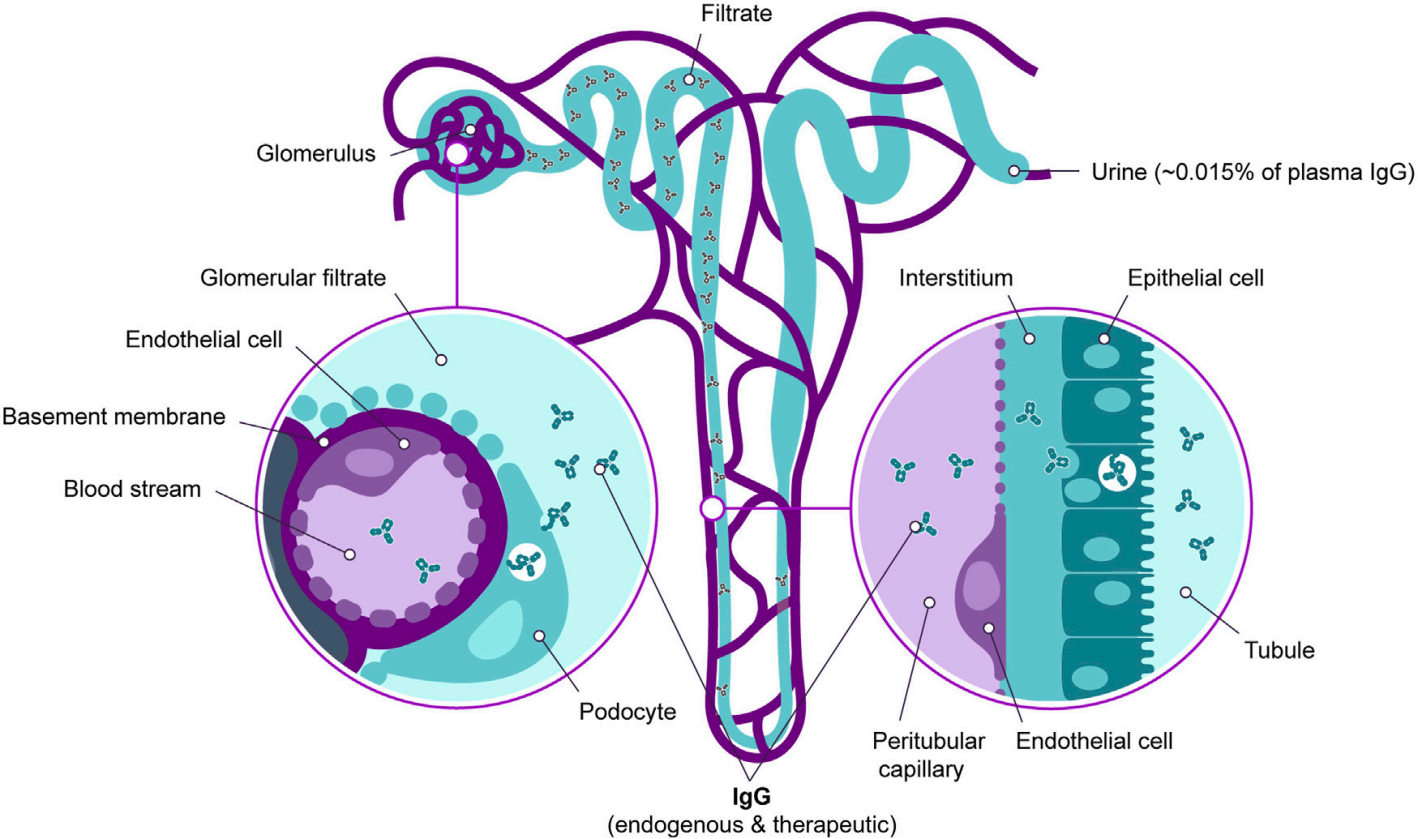
Passage of serum IgG through the kidney endothelial barrier. IgG in the bloodstream enters the glomerular filtrate and the tubular lumen. It is then recycled back into the bloodstream through specific transport mechanisms. Additionally, a significant fraction of IgG is secreted via the urine.

Overall, this data provides evidence that a small but non-negligible amount of serum IgG, whether of endogenous origin or therapeutically administered, passes the endothelial barrier and is detectable in the urine. This process is summarized in Figure 2 below.

Strategies aimed at enhancing transcytosis of therapeutic antibodies across the kidney endothelial barrier, such as leveraging receptors expressed on renal endothelial cells, have, to our knowledge, not been reported. Other options such as small antibody fragments or even smaller antibody-like molecules such as darpins might offer improved tissue penetration but carry the risk of shorter half-lives and impaired interaction with IgG-specific transcytosis mechanisms due to their inability to bind to the FcRn, potentially further decreasing transcytosis efficiency (Rafidi et al., 2022). Therefore, developing ultra-potent therapeutic antibodies that remain effective at low concentrations currently represents the most promising alternative to treat diseased or infected tissue that lies behind the kidney endothelial barrier.

While previous studies have established the existence of transport mechanisms for human IgG through the endothelial cells of blood vessels to the renal epithelia, as well as the reabsorption mechanisms from the urine by proximal tubular epithelial cells via specific transport mechanisms (McCarthy et al., 2000; Pyzik et al., 2019; Bryniarski et al., 2021), they did not provide quantitative data, and such data from preclinical animal models have also not been found in the literature.

Our observation that on average 0.015% of serum IgG makes its way into the urine may serve a basis for dose finding of therapeutic antibodies intended for activity in the kidney and urogenital tract.

However, a limitation of our study is that it cannot provide quantitative data on the antibody concentration in the lumen of tubular epithelia or the urinary ducts. Nevertheless, due to the reabsorption mechanisms, the IgG concentration in the tubular lumen and ureter can be assumed to be substantially higher than the determined urine-serum ratio of 0.015%.
