# Supplementary material for: Quantification of endogenous and therapeutic IgG crossing the kidney barrier from bloodstream to urine
Source: Front Pharmacol. 2025 Apr 25;16:1572739. doi: 10.3389/fphar.2025.1572739 (PMC12061720; doi:10.3389/fphar.2025.1572739)
Supplement: Supplementary file 1 [file DataSheet1.pdf]

Supplementary Material

Table S1 Donors´ characteristics

Donor	Primary Diagnosis	Gender	Race	Age	Height (cm)	Weight (kg)	Tobacco History	Alcohol History	Treatment Notes	Previous Treatments	Current Medications
1	NHL, Diffuse Large B-Cell Lymphoma	Male	White	74	184.4	84.82	Never Used	Current Use - Light	RCHOP	elitek	asa 81, fluorouracil, lidocaine-prilocaine, metoprol suc, prednisone, tamsulosin, udenyca
2	NHL, Follicular Lymphoma	Female	White	73	161.29	79.38	Never Used	No Use	Bendeka+Rituxan	none	calcium carbonate, compazine, fluoxetine, ondansetron hci, preservison AREDS, prevagen, protonix, rosuvastatin, sleep aid, trazadone, udenyca, xanax
3	NHL, Diffuse Large B-Cell Lymphoma	Female	White	71	165.1	74.84	Never Used	No Use	Pola-R-CHP	none	acyclovir, bactrim ds, carvedilol, eliquis, esomeprazole magnesium, fluconazole, jardiance, levothyroxine, lidocaine-prilocaine, olmesartan-amlodipine, ondansetron hci, prednisone, rosuvastatin, tolterodine er
4	NHL, Follicular Lymphoma	Male	White	53	187.96	91.63	Current Cigarette	Current Use - Heavy	Bendamustine+Rituxan	none	asa 81
5	NHL, Follicular Lymphoma	Male	White	77	175.26	104.33	Previous Use	Previous Use - Light	Rituxan	N/A	N/A
